# Postoperative socket irrigation with drinking tap water reduces the risk of inflammatory complications following surgical removal of third molars: a multicenter randomized trial

**DOI:** 10.1007/s00784-016-1751-1

**Published:** 2016-02-27

**Authors:** H. Ghaeminia, Th. J. M Hoppenreijs, T. Xi, J. P. Fennis, T. J Maal, S. J. Bergé, G. J. Meijer

**Affiliations:** 1 Department of Oral and Maxillofacial Surgery, Radboud University Medical Center, Geert Grooteplein-Zuid 10, 6525 GA Nijmegen, The Netherlands; 2 Department of Oral and Maxillofacial Surgery, Rijnstate Hospital Arnhem, Wagnerlaan 55, 6815 AD Arnhem, The Netherlands; 3 Oral and Maxillofacial Surgery, ZBC Private Clinic Nijmegen, Groenewoudseweg 315, 6524 TX Nijmegen, The Netherlands; 4 Implantology & Periodontology, Radboud University Medical Center, Phillips van Leydenlaan 25, 6525 EX Nijmegen, The Netherlands

**Keywords:** Prevention, Irrigation, Drinking water, Pain, Trismus, Quality of life, Oral health impact profile, Risk factors

## Abstract

**Objectives:**

The primary aim of the present study was to evaluate the effectiveness of postoperative irrigation of the socket with drinking tap water on inflammatory complications following lower third molar removal.

**Material and methods:**

A multicenter randomized controlled trial was carried out from June 2013 to June 2014. In one arm of the study, patients were instructed to irrigate the tooth socket and surgical site with a Monoject® Curved 412 Tip Syringe (Tyco/healthcare-Kendall, Mansfield, MA, USA) with tap water. In a second arm of the study, the standard postoperative instructions did not include irrigation instructions. The incidences of alveolar osteitis and wound infection were recorded for each group and analyzed by the Fisher’s exact test.

**Results:**

A total of 280 patients with 333 mandibular third molars were analyzed. According to the intention-to-treat (ITT) analysis, inflammatory complications occurred in 18 cases in the Monoject® group (11.4 %) compared to 34 cases (19.1 %) in the control group (*p* = 0.04). These complications were associated with significant worse outcomes regarding quality of life, pain, and trismus and caused significantly more missed days of work or study. Female gender, age >26, bone removal, deep impacted third molars, less experienced surgeons, and a high amount of debris at the surgical site were also identified as risk factors for developing inflammatory complications following lower third molar removal.

**Conclusion:**

Irrigation of the surgical site with drinking tap water using a curved syringe following removal of third molars is effective in reducing the risk of inflammatory complications.

**Clinical relevance:**

Water is a very accessible, cost-effective irrigant without side effects and the results from this study have proven that it can be used to reduce the risk of inflammatory complications and associated morbidity following lower third molar removal.

## Introduction

Surgical removal of third molars, as one of the most common procedures in oral and maxillofacial surgery, is often accompanied by pain, swelling, trismus, and oral dysfunction. In normal healing, the most of pain and swelling reduces within 2 or 3 days. However, wound healing may be delayed due to alveolar osteitis (AO) or wound infection at surgical sites [1]. These complications are accompanied by painful symptoms and a significant impact on the quality of life, resulting in loss of patient’s productivity and working day’s [2].

The most common complication following mandibular third molar removal is AO with a reported incidence of 1–37 % [3]. The causes of AO are not completely known, but the destruction of the blood clot by invading oral bacteria is generally accepted as an important etiological factor [3–5]. Following destruction of the thrombus, the socket may become packed with food remnants and debris leading to further disturbed wound healing [1, 3]. Various factors have been considered to be associated with an increased risk for developing AO, such as the female gender [6–9], smoking [10, 11], inadequate oral hygiene [9], surgical trauma [10, 12, 13], and removal of teeth with pre-existing infection or pathology [14].

Forty-five percent of patients with AO require multiple postoperative visits, which is not only an inconvenience for the patients but also a burden from financial perspectives [15]. Most common treatment for AO tends to focus on symptomatic relief, which includes the removal of debris from the tooth socket by irrigation with saline solution using a syringe [3] and prescription of analgesics. To support the oral hygiene in and around the tooth socket and to prevent inflammatory complications following surgical removal of lower third molars, some surgeons instruct the patient to irrigate the surgical site with drinking tap water using a syringe. Surprisingly, the efficacy of this simple non-invasive method has not been investigated yet.

The primary aim of the present study was to evaluate the effectiveness of postoperative irrigation of the surgical site with drinking tap water using a Monoject® syringe on postoperative complications following lower third molar removal. The secondary objective was to investigate the impact of wound infection and alveolar osteitis on quality of life measures and to identify risk factors associated with these complications.

## Material and methods

This RCT has been described according to the CONSORT statement 2010 (http://www.consort-statement.org/).

### Study design

This study was part of a multicenter randomized controlled clinical trial investigating the efficacy of cone beam CT (CBCT) prior to mandibular third molar removal from which the trial and the clinical protocol were approved by the Institutional Review Board (CCMO Arnhem-Nijmegen, NL nr.: 40492.091.12). All patients were informed about the study and a written informed consent was obtained. The study was performed in three participating departments of oral and maxillofacial (OMF) surgery of (1) Radboud University Medical Centre Nijmegen (RUN), (2) Rijnstate Hospital Arnhem (RHA), and a private clinic in Nijmegen (PCN).

### Participants

The procedure of selecting patients and eligibility criteria are described in detail in a previous article [16]. Prior to surgery, the patients characteristics were recorded at baseline in a secured website designed for this study.

### Surgery

All mandibular third molars were removed under local anesthesia without antibiotic prophylaxis or pre- and postoperative antiseptic rinses. Intra-operative variables, such as experience of the surgeon, duration of surgery, technique of third molar removal, number and shape of roots were registered through the website. All patients received a pain diary with a visual analogue scale (VAS) and validated Dutch version of Oral Health Related Quality of Life (OHIP-14) forms 1 day before until 7 days after surgery. A review appointment 7 days after surgery was made.

### Randomization

At the final stage of surgery, a surgical assistant assigned the patients randomly through a computer random generator after logging in the secured website. The allocation concealment was guaranteed through the Web-based central concealment.

Patients were allocated either to the following:

1. Monoject® syringe group. After surgery, a curved tip Monoject® syringe (12 cm^3^) was provided to the patient. In addition to the standard postoperative care instructions, the participants received instructions with regard to the use of Monoject® syringe (by bringing the tip at the distal side of the second molar in or above the tooth socket and irrigate four times a day with plain tap water). To avoid early removal of the blood clot, patients were instructed to start irrigating the wound 48 h after surgery until the first postoperative visit 7 days after surgery.
2. Standard postoperative care instructions, without the use of a Monoject® syringe. The standard postoperative instructions were biting on a gauze for 30 min, no rinsing and spitting for the first 24 h, and starting the regular tooth brushing the day after surgery. Paracetamol (4 times a day 1000 mg) in combination with ibuprofen (3 times a day 600 mg) were prescribed postoperatively.

### Outcomes

The primary outcome measures were the number of lower third molars with postoperative inflammatory complications, which included surgical wound infection and AO.

The secondary outcomes consisted of quality of life measures, including pain (VAS score), trismus (change in maximum interincisal distance), OHIP-14, number of emergency visits, and missed days of work or study.

One blinded investigator per center assessed the primary and secondary outcome measures.

Surgical wound infection was defined as the presence of a local abscess, onset of facial or cervical abscess/cellulitis, and other signs suggesting an infection (redness, swelling, purulent discharge, fever). The diagnosis of AO was based on the Blum criteria: postoperative pain in and around the extractions site, which increased in severity at any time between 1 and 3 days after the extraction, accompanied by a partially or totally disintegrated blood clot within the alveolar socket with or without halitosis [3]. A distinction was made in patients with more severe symptoms: irradiating pain, which was not adequately relieved with the standard analgesics.

**Fig. 1:**
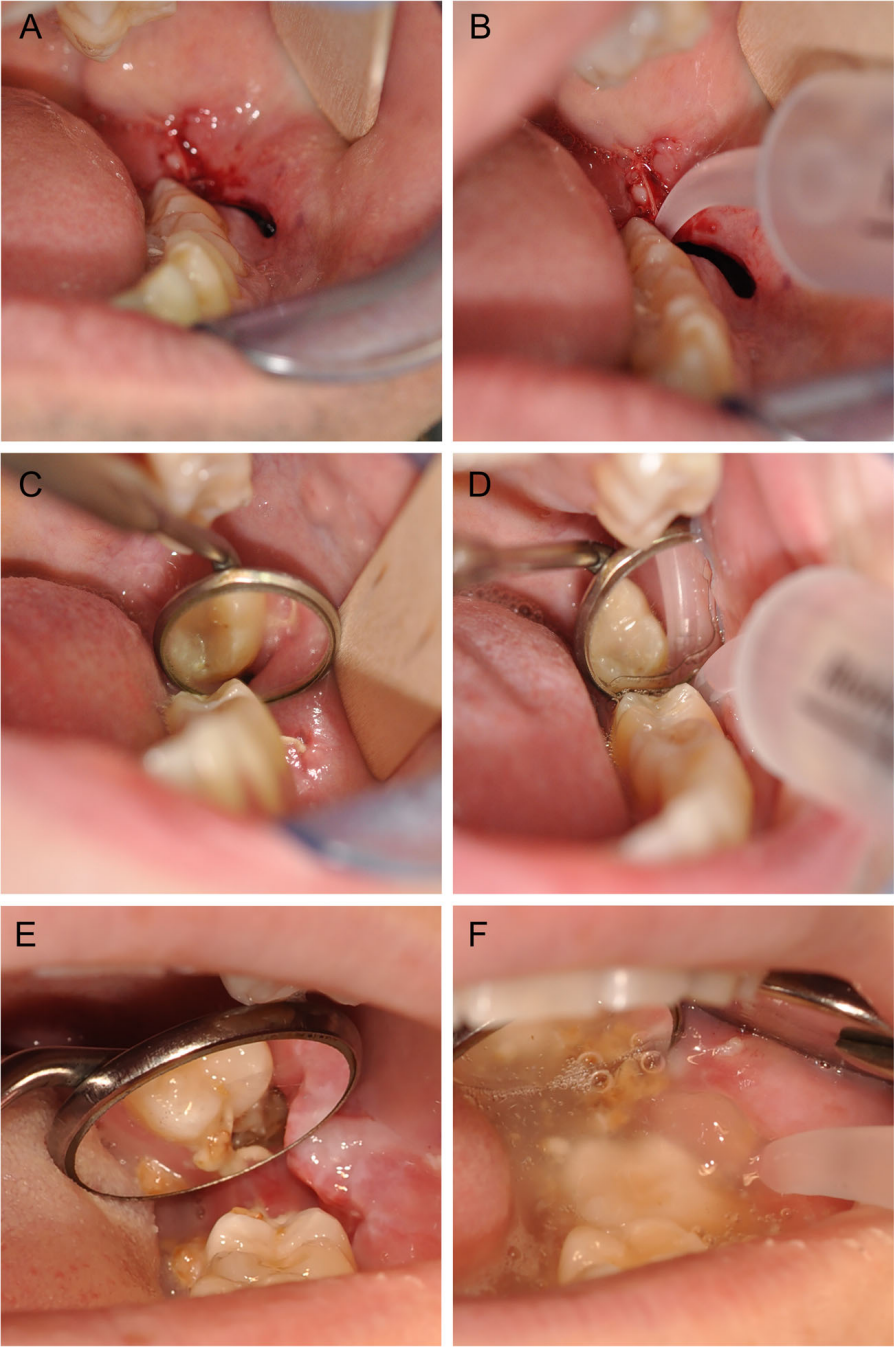
Irrigation of the surgical site with a curved tip Monoject® syringe. Surgical site immediately following the removal of the 38 (**a**). According to the web-based randomization, the patient was assigned into the Monoject® group, and instruction on the use of the syringe was provided (**b**). The surgical site (38) of a patient with normal healing 1 week after surgery (**c**). Following irrigation of the surgical site, no debris was found (**d**). A surgical site (38) of an alveolar osteitis (**e**). After irrigation, a high amount of debris was assessed (**f**)

After assessment and registration of the wound healing in the website, the surgical site was irrigated with sterilized water using a Monoject® syringe. The amount of debris in and around the alveolus was registered on a four-degree scale (Fig. 1).

Finally, the patient was asked to demonstrate how the Monoject® was used. If the patient failed to use the Monoject®, or if the Monoject® was not used according to the instructions (adequate irrigation by bringing the tip at the distal side of the second molar in or above the tooth socket), this was registered as well.

The number of postoperative visits and possible postoperative interventions such as wound irrigation, use of antibiotics, abscess incision, and drainage or exploration of the wound within 2 months were registered at the website.

### Statistical methods

The primary and secondary outcome measures were analyzed according to the intention-to-treat (ITT) and treatment received (TR) analyses. In the TR group, the protocol violations (patients not attending for the postoperative visit 1 week after surgery and surgical sites not being irrigated according to the instructions) were excluded from analyses. The means and standard deviations of normally distributed variables were calculated and analyzed by the independent-samples *t* test. Dichotomous variables were analyzed by the chi-squared or the Fischer’s exact test. Logistic regression analysis was performed to identify possible risk factors for inflammatory complications. Dependent variables in each analysis that was significant at the *p* < 0.05 were considered for the multivariate analysis. The unadjusted and adjusted odds ratios with 95 % confidence intervals (CIs) were estimated. The SAS® 9.2 was used for data analyses.

## Results

**Fig. 2:**
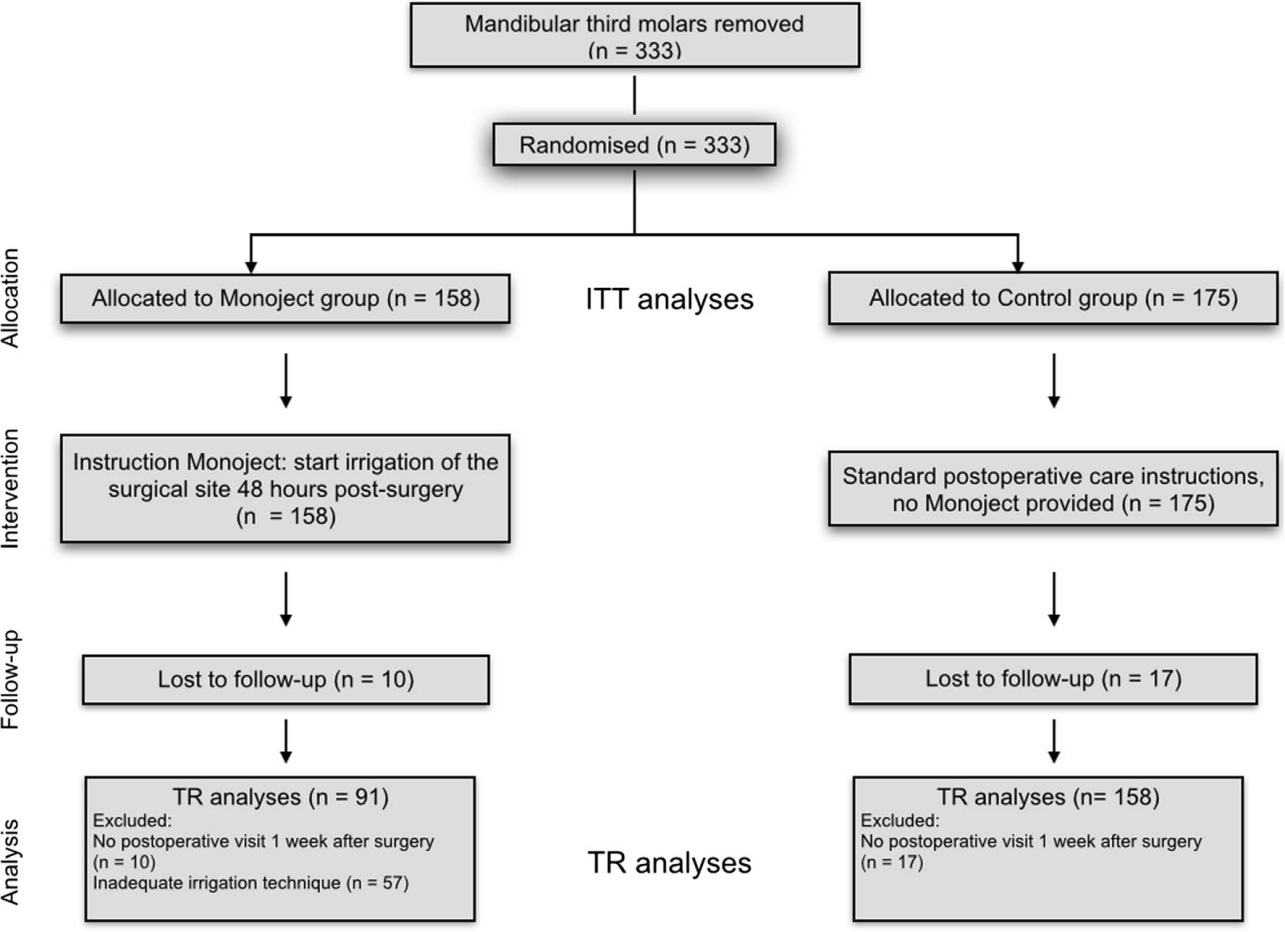
diagram with flow of patients (number of third molars). *n* number, *ITT* intention to treat, *TR* treatment received

Figure 2 represents the flow of 280 patients with 333 randomized third molars during the phases of the study regarding the ITT analysis and TR analyses. The inclusion of the three centers RUN, RHA, PCN resulted in 104, 111, and 65 third molars, respectively. The majority of the third molars were bony impacted (68 %), necessitating surgical bone removal (76 %).

In the Monoject® group, 67 of the 158 surgical sites (42.2 %) were not irrigated by the patient according to the instructions and were excluded for the TR analyses.

None of the baseline characteristics differed significantly between the two intervention groups for the ITT and TR analyses (Appendix Table 4).

The results of the primary outcomes are presented in Table 1. The overall incidence of inflammatory postoperative complications following third molar removal was 15.6 %. According to the ITT analysis, these complications developed in 18 cases in the Monoject® group (11.4 %) compared to 34 (19.1 %) in the control group, which is a significant difference (Fisher’s exact test, two-tailed, *p* = 0.04). This was primarily the result of a significant lower incidence of AO (*p* < 0.005) in the Monoject® group (5.5 %) compared to the control group (15.7 %). For the TR analyses, the incidence of inflammatory complications were 8.7 % for the Monoject® group and 20.9 % for the control group (*p* < 0.01).

**Table 1:** Primary outcome measures for ITT and TR analyses

	ITT analyses			TR analyses		
Primary outcome	Monoject® (*n* = 158)	Control (*n* = 178)	*p*	Monoject® (*n* = 91)	Control (*n* = 158)	*p*
Inflammatory complications	18 (11.4 %)	34 (19.1 %)	0.04*	8 (8.7 %)	33 (20.9 %)	0.01*
Alveolar osteitis	9 (5.7 %)	28 (15.7 %)	0.005*	5 (5.4 %)	27 (17 %)	<0.001*
Moderate symptoms	7 (4.5 %)	18 (10.1 %)	0.04*	4 (4.4 %)	17 (10.8 %)	0.09
Severe symptoms	2 (1.3 %)	10 (5.6 %)	0.04*	1 (1.1 %)	10 (6.3 %)	0.06
Wound infection	9 (5.7 %)	6 (3.4 %)	0.43	3 (3.3 %)	6 (3.8 %)	1.0

*ITT* intention to treat, *TR* treatment received

*Statistically significant difference (*p* < 0.05)

**Fig. 3:**
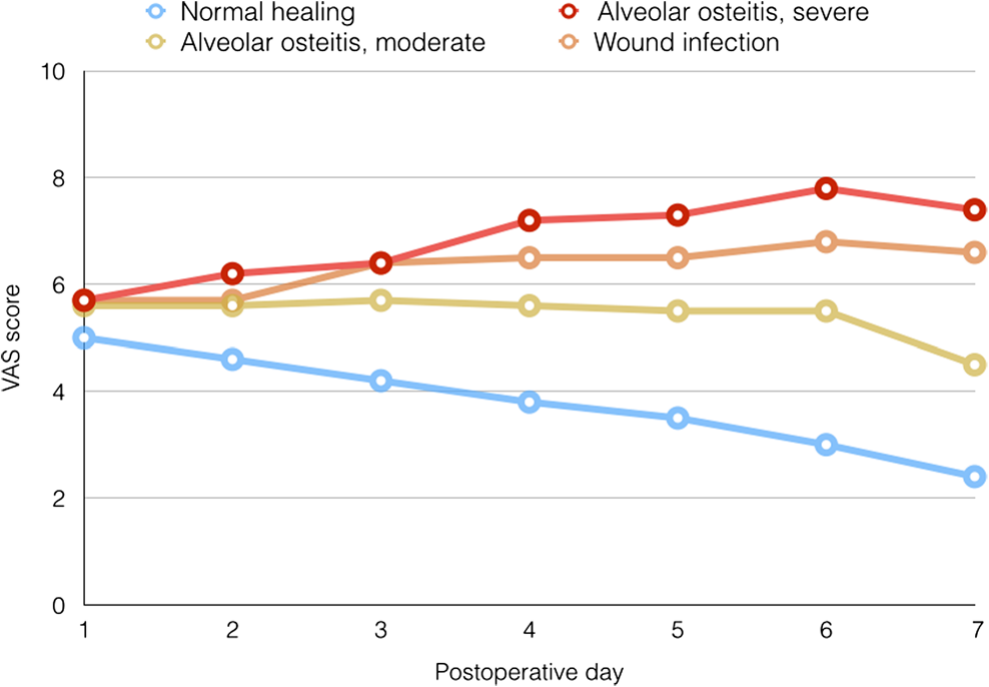
Pain scores from day 1 to day 7 after removal of the third molar for patients with normal healing and inflammatory complications

Patients with AO and surgical wound infections following third molar removal had significantly higher pain scores (*p* < 0.0001) and worse quality of life scores (*p* < 0.0001) for the first 7 postoperative days compared to patients without these complications (Table 2). The presence of these complications resulted in a reduced mean mouth opening of 18.2 mm compared to a mean reduction of 8.3 mm in cases of normal healing 1 week after surgery. Patients proceeded with work or study after a mean period of 1.7 days in case of normal healing compared to a mean period of 3.3 days in case of inflammatory complications (*p* = 0.01) (Fig. 3).

**Table 2:** Effect of inflammatory complications on pain, quality of life, trismus, and number of missed days of work or study

	Inflammatory complications (*n* = 52)	No inflammatory complications (*n* = 281)	*p*
Pain (VAS score)	6.0 ± 1.9	3.8 ± 2.0	<0.0001*
Day 1	5.6 ± 2.1	5.0 ± 2.5	<0.0001*
Day 2	5.8 ± 2.2	4.6 ± 2.5	<0.0001*
Day 3	6.1 ± 2.2	4.2 ± 2.4	<0.0001*
Day 4	6.3 ± 2.4	3.8 ± 2.4	<0.0001*
Day 5	6.3 ± 2.3	3.5 ± 2.3	<0.0001*
Day 6	6.5 ± 2.3	3.0 ± 2.2	<0.0001*
Day 7	5.9 ± 2.4	2.4 ± 2.2	<0.0001*
OHIP-14 (days 1–7) [0–56] (mean ± s.d.)			
Functional limitation [0–8]	2.9 ± 2.3	1.3 ± 1.4	<0.0001*
Physical pain [0–8]	5.8 ± 2.4	4.5 ± 2.3	0.0002*
Psychological discomfort [0–8]	4.2 ± 2.3	1.9 ± 1.8	<0.0001*
Physical disability [0–8]	4.9 ± 2.5	3.1 ± 2.2	<0.0001*
Psychological disability [0–8]	3.1 ± 2.2	1.4 ± 1.6	<0.0001*
Social disability [0–8]	4.2 ± 2.5	2.3 ± 1.9	<0.0001*
Handicap [0–8]	3.7 ± 2.8	1.5 ± 1.9	<0.0001*
Change in IID in mm (mean ± s.d.)	−18.2 ± 11.8	−8.3 ± 11.3	<0.0001*
Number of missed days of work or study (mean ± s.d.)	3.3 ± 3.9	1.7 ± 1.9	0.01*

*N* number of mandibular third molars, *VAS* visual analogue scale (range 1–10), *OHIP*-*14* Oral Health Impact Profile 14, *IID* interincisal distance, *s*.*d*. standard deviation

*Statistically significant difference (*p* < 0.05)

### Risk factors

Multivariate regression analysis demonstrated that female gender (OR 5.6, 95 % CI 2.2–14.4, *p* < 0.001), high amount of debris at surgical site (*p* < 0.001), age >26 years (*p* = 0.04), resident surgeons (*p* < 0.02), bone removal (*p* = 0.03), and class III depth of impaction (*p* = 0.04) were significantly associated with inflammatory complications following mandibular third molar removal (Table 3).

**Table 3:** Risk factors for inflammatory complications following surgical removal of third molars

			Unadjusted		Adjusted	
	I	NI	OR (95 % CI)	*p*	OR (95 % CI)	*p*
Variables						
Age						
18–25 years (C)	29	199				
>26 years	23	81	*1.95* (*1.06–3.57*)	0.031	*2.13* (*1.04–4.36*)	0.037
Gender						
Male (C)	7	105				
Female	45	175	*3.86* (*1.68–8.87*)	0.0015	*5.59* (*2.17–14.41*)	0.0004
Oral contraceptive drugs						
Yes	25	112				
No	15	55	1.22 (0.59–2.50)	0.58	0.99 (0.45–2.18)	0.98
Unknown	5	8				
Oral hygiene						
Good (C)	41	233				
Moderate	2	23	0.49 (0.11–2.18)	0.35	0.55 (0.12–2.61)	0.45
Poor	2	0	NE	0.99	NE	0.99
Smoking >3 days (yes)	8	33	1.34 (0.58–3.10)	0.50	1.65 (0.62–4.39)	0.31
Pericoronitis (yes)	10	63	0.83 (0.39–1.77)	0.63	1.33 (0.55–3.21)	0.53
Pocket >4 mm + bleeding (yes)	17	87	1.09 (0.57–2.08)	0.81	1.03 (0.47–2.25)	0.95
Surgical variables						
Experience of the surgeon						
Senior (C)	22	165				
Resident	29	116	*1.88* (*1.03–3.43*)	0.041	*2.20* (*1.11–4.33*)	0.024
≥3000 M3 removed (C)	36	203				
<3000 M3 removed	15	78	1.08 (0.56–2.09)	0.81	1.07 (0.50–2.29)	0.85
Duration of surgery	12.7	11.3	1.02 (0.99–1.06)	0.27	0.99 (0.95–1.05)	0.90
Bone removal (yes)	45	208	*2.60* (*1.06–6.34*)	0.036	*2.86* (*1.08–7.56*)	0.034
Incision						
Envelope (C)	15	79				
Triangular	35	178	1.03 (0.54–2.00)	0.92	1.05 (0.49–2.25)	0.90
Other	0	19	NE		NE	
Technique of closure						
Complete closure (C)	18	87				
Opening from occlusal	19	126	0.73 (0.36–1.47)	0.38	1.01 (0.45–2.28)	0.98
Opening from mesial	13	60	1.05 (0.48–2.30)	0.91	1.80 (0.69–4.72)	0.23
Number of sutures						
1 (C)	1	24				
2	18	107	4.03 (0.51–31.7)	0.18	2.61 (0.27–25.4)	0.41
3	26	124	5.03 (0.65–38.9)	0.12	2.87 (0.29–28.5)	0.37
>3	5	18	6.67 (0.72–62.1)	0.10	4.48 (0.36–55.6)	0.24
Anatomical variables						
Depth of impaction^a^						
Tooth covered by anterior border of the ramus						
Class I (C)	17	97				
Class II	27	173	0.89 (0.46–1.72)	0.73	0.87 (0.41–1.88)	0.88
Class III	8	10	*4.57* (*1.58–13.2*)	0.005	*3.77* (*1.07–13.3*)	0.039
Depth of impaction to the adjacent tooth						
Class A (C)	8	63				
Class B	31	185	1.32 (0.58–3.02)	0.52	0.85 (0.33–2.17)	0.73
Class C	13	32	*3.20* (*1.20–8.51*)	0.02	2.09 (0.67–6.56)	0.20
Other						
Amount of debris in alveolus						
None (C)	15	113				
Low	12	75	1.21 (0.53–2.72)	0.65	1.47 (0.62–3.48)	0.39
Moderate	9	32	2.12 (0.85–5.29)	0.11	2.33 (0.84–6.43)	0.11
High	14	26	*4.10* (*1.74–9.43*)	0.001	*4.87* (*1.91–12.4*)	0.0009

Italic values indicate statistical significance (*p* < 0.05). The odds ratios with their 95 % confidence intervals (CIs) were estimated by logistic regression models. The adjusted odds ratios with their 95 % CIs were estimated by multiple logistic regression models after controlling for Monoject®, age, gender, bone removal, depth of impaction to the adjacent tooth, and amount of debris in alveolus

*n* number of mandibular third molars, *VAS* visual analogue scale (range 1–10)

*Statistically significant difference (*p* < 0.05)

^a^Pell and Gregory classification

## Discussion

The results from this study have confirmed that inflammatory complications following the surgical removal of mandibular third molars are associated with significant morbidity and reduced quality of life, which is in line with other studies [2, 17, 18]. This resulted in an increase of the number of postoperative visits and missed days of work or study compared to patients without these complications.

Many efforts have been made in order to reduce the complications and associated morbidity following removal of lower third molars. Various surgical techniques [19–22], pre- and postoperative chlorhexidine rinses [23–25], local and systemic antibiotics [26–30], and a variety of intra-socket preventive and therapeutic measures have been developed and investigated [15]. Data from systematic reviews suggest only a slight benefit in reducing the risk of AO when a triangular flap is performed compared to an envelope flap [31], pre- and postoperative rinsing with chlorhexidine [15], and the use of prophylactic antibiotics [26]. The increase of adverse effects and bacterial resistance, however, does not favor the standard use of prophylactic antibiotics [26, 27].

It is remarkable that among all these preventive measures, the use of water to reduce complications following surgical removal of third molars has not been investigated yet. Water has the major advantage of being accessible and very cost-effective with no adverse effects. To our knowledge, this is the first study that has proven the effectiveness of postoperative irrigation of the socket with drinking tap water in reducing the risk of inflammatory complications following surgical removal of third molars. Cleaning of surgical wounds with water is an old and common procedure to prevent infections in extremities and drinking tap water is thought to be as good as saline or sterilized water for this purpose [32]. Recently, the first RCT was published investigating the effect of saline mouth rinse on postoperative complications following routine dental extractions [33]. A significant lower incidence of AO was found in the saline rinsing group compared to no rinsing. In this study, all subjects used prophylactic antibiotics and only healthy patients acquiring non-surgical routine dental extractions where included. Therefore, these results cannot be compared with the results from the present study. Mere rinsing without the use of a syringe, in case of surgical removal of the third molar, might be less effective to adequately clean the surgical site, due to the dorsal position in the dental arch in combination with trismus often accompanied with these procedures. This is supported by the TR analyses in the present study: the risk of AO was lower if the Monoject® syringe was used adequately. It is worth mentioning that a significant number of patients failed to use the syringe according to the instructions, regardless of the educational level of the patient. This emphasizes the need for additional methods to provide postoperative care information, such as the use of animations on websites or applications for smartphones.

An important strength of this study was the Web-based randomization and data entry. This minimizes the risk of selection and attrition bias and enlarges the possibilities to register a wide range of patient characteristics in a prospective way. The baseline characteristics and possible risk factors for postoperative complications were therefore very well balanced between both study arms. Another strength was that the study was performed in three different settings (university clinic, public hospital, and private clinic) with different surgeons being educated in different centers, which implies good generalizability. The selection of patients and calculation of the sample size was based on a randomized clinical trial investigating the usefulness of cone beam CT (CBCT) in patients with an increased risk for inferior alveolar nerve injury following the removal of mandibular third molars, which might be a potential weakness in this part of the study. Since subjects with a pre-operative CBCT were evenly distributed between both study arms in this part of the study, it can be expected that this co-intervention did not influence the results. Furthermore, a pre-operative CBCT had no influence on the outcome of postoperative complications, pain, quality of life, and duration of surgery [16]. Due to the selection criteria of the CBCT study, mainly third molars with deep impactions where included. It should be emphasized, that the results of this study are not applicable for non-surgical extractions. Another potential weakness is that the frequency and dosage of the prescribed analgesics were not registered appropriately in the pain diaries of the patients. Therefore, it was not possible to correlate the VAS scores with the actual used analgesic drugs.

To date, there is no consensus regarding the diagnostic criteria and terminology for AO used in the literature, which explains the great variability in the reported incidences of 1–37 % following third molar removal [3, 4, 15]. Traditionally, the condition was defined as an empty tooth socket with exposed bone, accompanied with a continuous severe irradiating pain [34]. More recent studies [15, 35, 36] use the definition of AO according to Blum [3], which also includes a partially empty tooth socket and furthermore makes no distinction in the type and severity of pain. To allow comparability with results from other studies, the definition of AO according to Blum was used in the present study. Using these criteria, the incidence of AO following third molar removal is reported to be between 25–30 % [3], which is higher than the overall incidence of AO of 11 % in the present study. Most patients seen on emergency visits with painful symptoms following surgical removal of third molars have met the criteria of AO according to Blum, in which the clinical assessment showed a partially disintegrated blood clot in the tooth socket filled with debris remnants. Irrigation of the socket and continuing the regular analgesics are usually sufficient in these cases. However, in our experience, patients with a true dry socket have a different clinical presentation with a severe irradiating pain, usually necessitating stronger analgesics and more postoperative visits. From this point of view, it is important to distinguish these clinical entities, and therefore, a distinction was made in a moderate and severe presentation of AO in the present study.

Although the pathogenesis is not completely known, an increased fibrinolysis in the blood clot is thought to be the major contributing factor for AO [3–5]. Birn has extensively studied the pathogenesis of AO and found an increased fibrinolytic activity as well as activation of plasminogen into plasmin, in the presence of tissue activators in dry sockets [37]. He stated that these tissue activators are released after trauma to the alveolar bone or elaborated by bacteria, resulting in disintegration of the blood clot [12]. The multivariate regression analysis in this study has proved that surgical removal of the bone, deep impactions, and less experienced surgeons were independent significant risk factors for inflammatory processes. These factors indicate a more traumatic tooth removal resulting in more obvious postoperative complications, which has also been demonstrated in previous studies [5, 10, 12–14, 38]. Beside surgical trauma, bacterial invasion was suggested to play an important role in the development of AO and postoperative wound infection [3, 10, 39, 40]. Blum stated that despite a lack of scientific evidence, it seems logical that fragments and debris could lead to a disturbed wound healing and thereby possibly contribute to the development of an AO [3]. The results from this study showed a strong significant association between high amount of debris remnants at the surgical site and inflammatory complications. This underlines that debris remnants should be regarded as one of the contributing factors for AO. The low incidence of AO in the Monoject® group of 5.5 % is probably the result of effective mechanical removal of debris, bacterial colonization, and associated metabolic wastes within the tooth socket.

It has been postulated that direct excessive irrigation of the alveolus might wash out the blood clot and thereby increase the risk of AO [41]. Although this seems plausible, sound evidence to support this theory is lacking [3, 5, 42]. In the authors view, excessive intra-alveolar irrigation at the first day following surgery should be avoided. Theoretically, one might start the irrigation before the internal dissolution of the blood clot occurs. Birn stated that the increase in fibrinolysis is unlikely to dissolve the blood clot before the second day after surgery, since the clot contains antiplasmin, which must be neutralized before clot dissolution can occur [4]. Therefore, the best moment to start the irrigation might be somewhere between the first and second postoperative day. Nevertheless, the results from this study prove that wound irrigation starting 48 h after surgical removal of the third molars is a safe procedure to perform. To prevent AO, it has been postulated to apply topical antifibrinolytic agent tranexamic acid in the tooth socket. A randomized controlled trial performed in 1979 did, however, not show a significant reduction in the incidence of AO when compared with placebo following removal of the third molars [43].

Increasingly, patients request personalized information about their risks and potential benefits of removing a third molar. Although the risk for inflammatory complications following surgical removal of the third molars is multifactorial in nature, identifying risk factors will aid to inform the patients more accurately about these anticipated complications. Increasing age is a well-known risk factor for complications following third molar removal [2, 44–46], which has been confirmed in the present study. This might influence the decision-making process whether or not to remove an asymptomatic third molar at a younger age [47]. Another important risk factor found in this study was the female gender. The odds of developing inflammatory complications are five times higher for female patients compared to male patients. The increased risk for female patients is found in several studies [2, 6–9], while others did not find this association [44]. It is suggested that the higher incidence of AO in female patients is caused by the use of oral contraceptives. Estrogen in oral contraceptives has shown to cause elevated plasma fibrinolytic activity [48], which could in turn cause earlier lysis of the blood clot [3–5]. The multivariate regression analysis in this study, however, did not show any effect of the use of oral contraceptives on the occurrence of postoperative complications following third molar removal. Furthermore, no relationship was observed between smoking, oral hygiene, and inflammatory complications as was demonstrated in previous studies [9–11], probably due to a low incidence of smokers and a very low incidence of patients with a poor pre-operative oral hygiene in our study population.

## Conclusion

Postoperative inflammatory complications following removal of third molars has a significant impact on the quality of life of patients, resulting in increased missed days of work and study. Female gender, increasing age, deeply impacted mandibular third molar, bone removal, less experienced surgeons, and debris remnants in and around the tooth socket were associated with an increased risk to develop these postoperative complications. The risk of alveolar osteitis following surgical removal of mandibular third molars can be significantly reduced by postoperative irrigation with plain drinking tap water. Starting 48 h after surgery, using a curved tip Monoject® syringe and rinsing four times a day during 5 days seems to be an effective protocol for this commonly performed surgical procedure. Special care should be provided on the postoperative instructions how to use the syringe.

## Appendix

**Table 4:** Baseline characteristics for intention-to-treat analyses and treatment received analyses

	ITT analyses			TR analyses		
	Monoject® (*n* = 158)	Control (*n* = 175)	*p*	Monoject® (*n* = 91)	Control (*n* = 158)	*p*
Demographic variables						
Age			0.88			0.76
18–25	109	119		64	107	
26–35	36	46		17	42	
36–45	9	7		7	6	
46–55	2	1		2	1	
56–65	1	1		0	1	
>65	0	1		0	1	
Gender			0.25			0.58
Female	99	121		59	109	
Male	58	54		31	49	
Race			0.83			0.42
Caucasian	137	140		86	140	
Other	10	12		4	12	
Education level			0.25			0.39
Primary education	15	8		7	8	
Secondary vocational (MBO)	51	49		31	49	
Higher professional (HBO)	51	53		36	53	
University	29	41		16	41	
Health status variables						
ASA classification			0.052			0.047
1	130	158		73	143	
2 or >2	27	17		17	15	
Diabetes mellitus (yes)	1	1	1.0	0	1	1.0
Immune deficiency (yes)	0	0		0	0	
Other chronic condition, medical treatment (yes)	8	5	0.39	4	4	1.0
Oral contraceptive drugs (yes)	57	80	0.30	34	71	0.48
Current smoking (yes)	18	23	0.74	11	20	1.0
Current alcohol (>3 days)	3	0	0.10	2	0	0.13
Oral hygiene			0.35			0.41
Good	124	150		80	137	
Poor	13	12		4	11	
Bad	0	2		0	2	
Anatomic variables						
Angulation of the third molar^a^			0.69			0.68
Vertical	49	54		30	50	
Disto-angular	10	18		6	17	
Mesio-angular	75	79		41	70	
Horizontal	21	20		12	17	
Transverse	1	3		0	3	
Depth of impaction^b^						
Tooth covered by anterior border of the ramus			0.35			0.16
Class I	59	55		39	52	
Class II	90	110		47	96	
Class III	9	9		5	9	
Depth of impaction to the adjacent tooth			0.16			0.067
Class A	28	43		14	37	
Class B	107	109		59	99	
Class C	23	22		18	21	
Surgical variables						
Experience surgeon			0.48			0.43
<100 M3 removed	5	11		3	11	
101–500 M3 removed	14	16		7	16	
501–1000 M3 removed	16	16		9	14	
1001–3000 M3	7	8		6	7	
>3000 M3 removed	116	123		66	109	
Resident	68	77	0.83	39	70	0.89
Senior staff	90	97		52	87	
Type of incision			0.35			0.08
Envelope	45	49		25	44	
Triangular	104	109		62	98	
Other	6	13	0.24	1	12	
Removal of buccal bone (yes)	125	128		74	113	0.09
Number of roots			0.93			
1	20	21		13	20	0.77
2	116	130		65	116	
>2	18	18		9	16	
Shape of roots			0.47			
Conical	30	24		18	23	0.42
Straight	62	81		32	71	
Curved	56	58		34	52	
Incomplete root formation	6	7		3	7	
Sectioning crown/roots (yes)	107	109	0.41	59	97	0.68
Sutures			0.51			0.29
1	12	13		6	11	
2	64	61		40	56	
3	69	81		38	71	
>3	9	14		3	14	
Technique of closure						
Complete closure	51	54	0.23	23	47	0.26
Opening from occlusal	74	71		48	67	
Opening from mesial	29	44		16	38	
Mean duration of surgery (min)	12.2	10.9	0.15	11.5	10.9	0.57
Pathological variables						
Pericoronitis (yes)	36	37	0.59	21	34	0.75
Pocket >4 mm + bleeding (yes)	50	54	0.81	29	51	1.0
Caries	2	9	0.07	1	9	0.10
Other						
CBCT pre-surgery	69	94	0.08	42	87	0.19
VAS pre-surgery (mean ± s.d.)	1.1 ± 2.2	1.1 ± 2.2	0.74	1.0 ± 2.2	1.1 ± 2.2	0.95
OHIP-14 pre-surgery (mean ± s.d.)	3.8 ± 3.8	4.1 ± 4.8	0.80	3.6 ± 3.8	4.2 ± 4.8	0.86
IID pre-surgery (mm) (mean ± s.d.)	47.3 ± 6.2	46.6 ± 7.6	0.42	47.5 ± 6.2	46.8 ± 7.6	0.49

*Statistically significant difference (*p* < 0.05)

^a^Winters classification

^b^Pell and Gregory classification

*n* number of mandibular third molars, *ASA* American Society of Anaesthesiologists, *CBCT* cone beam computed tomography, *VAS* visual analogue scale (range 1–10), *OHIP*-*14* Oral Health Impact Profile 14, *IID* interincisal distance, *s*.*d*. standard deviation
